# Accurate Identification of Protein Binding Sites for All Drug Modalities Using ALLSites

**DOI:** 10.1002/advs.202516530

**Published:** 2025-12-27

**Authors:** Minjie Mou, Mingkun Lu, Zhimeng Zhou, Yanlin Ren, Xinyuan Yu, Ziqi Pan, Yuan Zhou, Hao Yang, Lingyan Zheng, Shukai Gu, Yang Zhang, Wei Hu, Fengcheng Li, Haibin Dai, Feng Zhu

**Affiliations:** ^1^ Department of Pharmacy The Second Affiliated Hospital Zhejiang University School of Medicine Hangzhou China; ^2^ College of Pharmaceutical Sciences State Key Laboratory of Advanced Drug Delivery and Release Systems Zhejiang University Hangzhou China; ^3^ School of Pharmacy Hebei Medical University Shijiazhuang China; ^4^ Children's Hospital Zhejiang University School of Medicine National Clinical Research Center for Child Health Hangzhou China

**Keywords:** binding site, drug modality, protein druggability, protein language model, transformer

## Abstract

Proteins interact with diverse molecular modalities, yet the incomplete identification of their binding sites has left the proteome‐wide druggability largely underexplored. Although various computational methods have been developed for the prediction of protein binding sites, existing approaches are limited by their specificity to a single drug modality, dependence on high‐quality structural data, or insufficient predictive accuracy. Here, a unified sequence‐based framework, ALLSites, is constructed to identify proteome‐wide binding sites across all drug modalities. Leveraging ESM‐2 embeddings, ALLSites integrates a gated convolutional network with a transformer architecture to capture both global and local sequence features, effectively modeling residue interactions directly from sequence. This design bridges the gap between sequence‐based and structure‐based approaches, enabling ALLSites to achieve superior predictive performance across diverse drug modalities, including proteins, peptides, small molecules, carbohydrates, DNA, and RNA. It achieves state‐of‐the‐art performance among sequence‐based methods and matches the accuracy of the best structure‐based tools. By enabling accurate and structure‐free binding site prediction across all drug modalities, ALLSites is expected to expand the druggable proteome and provide a powerful resource for drug discovery.

## Graphical abstract

ALLSites is a unified sequence‐based framework for identifying proteome‐wide binding sites across all drug modalities. It integrates a gated convolutional network with a transformer architecture to capture residue interactions directly from the sequence. This design bridges the gap between sequence‐based and structure‐based approaches, enabling ALLSites to achieve superior predictive performance across diverse drug modalities, including proteins, peptides, small molecules, carbohydrates, DNA, and RNA. The balance between accuracy and applicability makes ALLSites a valuable resource for advancing the understanding of proteome‐wide druggability.

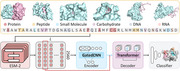

## 1 Introduction

Proteins play fundamental roles in cellular processes by interacting with a variety of molecular modalities [1, 2, 3]. Nevertheless, the druggability of proteins remains largely underexplored due to limited ligand‐modulated proteins and incomplete mechanistic understanding, as evidenced by small molecules’ ability to modulate less than 15% of the human proteome despite being the most prevalent drug modality [4, 5, 6, 7]. Researchers then began to explore alternative drug modalities, including protein‐, peptide‐, nucleic acid‐, and carbohydrate‐based therapeutics, to modulate protein functions [8, 9, 10, 11, 12]. Therefore, the comprehensive identification of binding sites for all drug modalities is of vital importance, as it can greatly expand proteome druggability by redefining “undruggable” proteins under one modality as “druggable” under another [13, 14, 15]. The diversity and complexity of drug modalities pose great challenges in the experimental identification of protein binding sites [16, 17, 18]. In response, substantial efforts have been devoted to developing computational methods for predicting protein binding sites across various drug modalities [19, 20, 21, 22].

Computational methods generally fall into two categories according to their input requirements, namely, structure‐based and sequence‐based ones [23]. For example, methods such as DeepPPISP [24], DELPHI [25], and EnsemPPIS [26] utilize either protein structural or sequence information to identify protein‐protein interaction (PPI) sites. Tools like PepBind [27] and PepSite [28] are specifically designed to predict peptide‐protein interaction (PepPI) sites. These tools facilitate the design of protein‐ and peptide‐based therapeutics. Several tools have been developed for predicting small‐molecule‐protein interaction (SMPI) sites. For example, P2Rank employs machine learning to identify potential small molecule binding pockets [29], while methods such as CAPSIF: V are tailored for predicting carbohydrate‐protein interaction (CarbPI) sites [30]. In addition, various nucleic acid‐binding site prediction tools have been developed to accelerate the understanding of biological processes and facilitate nucleic acid‐based drug design [31]. Examples include DNAPred [32] and DNABind [33] for predicting DNA‐protein interaction (DPI) sites, and NucleicNet for identifying RNA‐protein interaction (RPI) sites [34].

However, existing approaches still face challenges in accurately identifying and distinguishing binding sites across all drug modalities at the proteome‐wide scale. First, existing methods face issues of high dependency on precise structure or low predictive accuracy [26]. Specifically, the application of structure‐based methods is limited by the low availability of high‐resolution structures and high sensitivity to structural errors [35, 36]. In particular, many undruggable proteins lack experimentally determined structures, and the use of predicted structures may reduce the accuracy of structure‐based methods [37, 38]. By contrast, sequence‐based methods offer broader applicability but suffer from suboptimal predictive performance due to the neglect of residue interaction information [39]. Second, most existing methods are designed for modality‐specific binding site prediction, and there remains no universal method capable of predicting binding sites for all drug modalities [40]. Currently, only a few methods support the prediction of binding sites for multiple modalities. For example, GraphBind is tailored for nucleic acid (DNA/RNA) binding sites [41], while PepBCL is effective only for peptides and performs poorly on DNA or RNA [42]. Overall, the insufficient proteome/modality coverage and poor performance of existing methods hinder their practical application. Therefore, it is highly demanded to develop a method capable of accurately predicting proteome‐wide binding sites for all drug modalities.

In this study, considering that the key features of binding sites across various drug modalities is inherently encoded within the protein sequence, a unified sequence‐based framework, named ALLSites, is constructed to identify proteome‐wide binding sites for all drug modalities. Built upon the protein language model (PLM) ESM‐2 for sequence embedding, ALLSites integrates a gated convolutional network (GatedCNN) with a transformer architecture to jointly learn the global sequence features and local contextual patterns. Moreover, it can model complex residue interactions directly from sequence data, thereby bridging the gap between sequence‐based and structure‐based methods. ALLSites achieves accurate protein binding site prediction for various drug modalities, including proteins, peptides, small molecules, carbohydrates, DNA, and RNA, while maintaining broad applicability. It exhibits state‐of‐the‐art performance on these drug modalities, outperforming all sequence‐based methods and achieving results comparable to the best structure‐based method. The balance between accuracy and applicability makes ALLSites a valuable resource for advancing the understanding of proteome‐wide druggability and accelerating the translation of various molecular modalities into clinical applications.

## 2 Results and Discussion

### 2.1 The Framework of ALLSites for Predicting Binding Sites of All Drug Modalities

**FIGURE 1:**
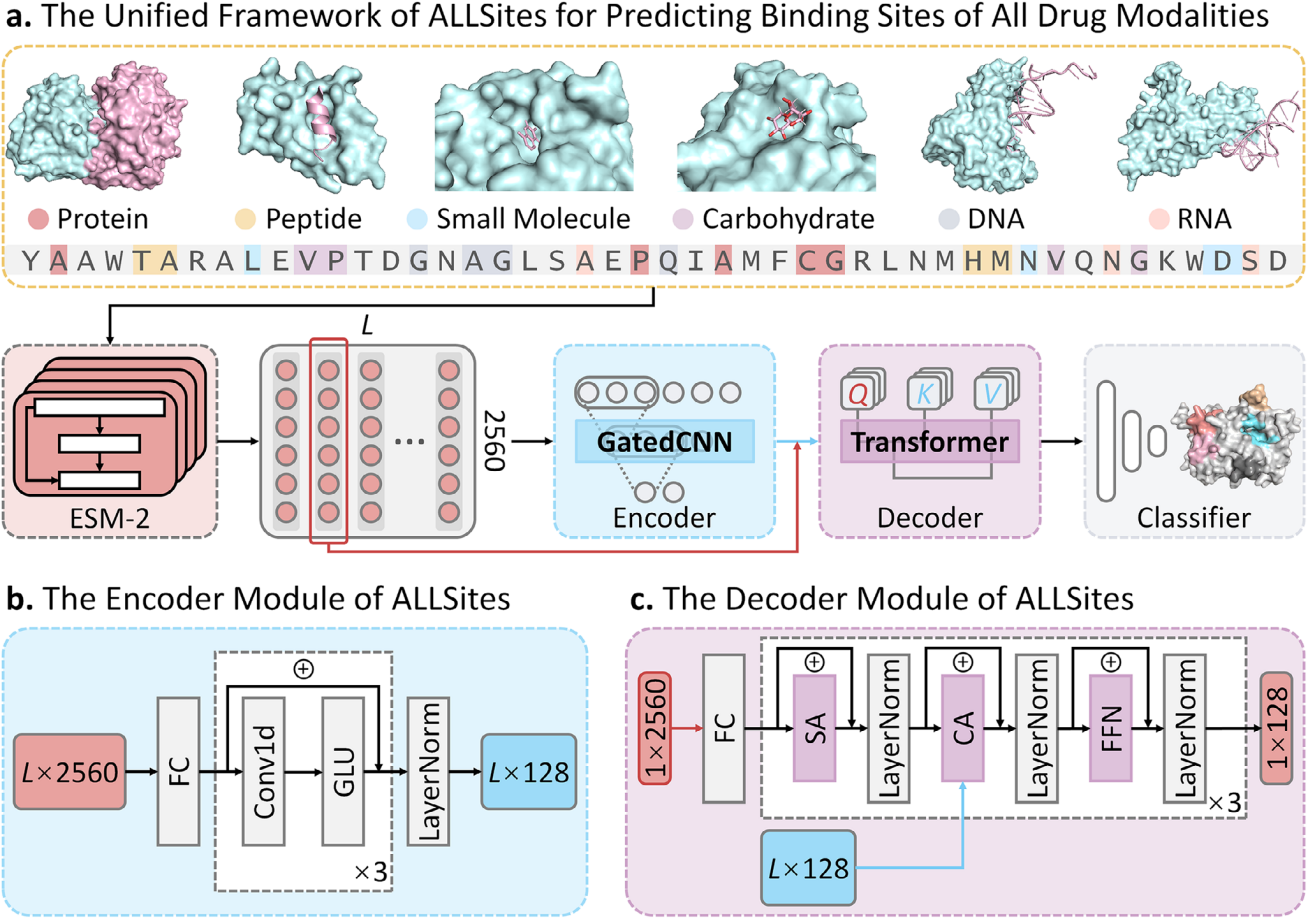
The framework of ALLSites for predicting protein binding sites of all drug modalities. a). The overall framework of ALLSites. Using only the protein sequence as input, ALLSites enables binding site prediction for multiple drug modalities, including proteins, peptides, small molecules, carbohydrates, DNA, and RNA. The model architecture comprises three main modules, namely a protein feature encoder for processing ESM‐2‐derived representations, a cross‐attention decoder, and a classification module. b). The architecture of the encoder module. The encoder is essentially a gated convolutional network (GatedCNN) designed to extract both local and global features from protein sequences. It consists of 1D convolutional layers (Conv1D) and the gated linear unit (GLU) activation function. c). The architecture of the decoder module. The decoder takes residue‐level representation and global protein feature as input. It employs a cross‐attention mechanism to learn residue interaction information. Both the encoder and decoder consist of three layers. FC, fully connected layers; SA, self‐attention; CA, cross‐attention; FFN, position‐wise feed‐forward network.

To accurately identify protein binding sites for all drug modalities, a unified deep learning framework named ALLSites is designed based on the transformer architecture. The overall framework of ALLSites is illustrated in Figure 1a. First, the model takes the protein sequence as input and employs the powerful PLM, ESM‐2, to generate residue‐level embeddings. Next, these embeddings are fed into an encoder that incorporates a GatedCNN, as shown in Figure 1b. The function of the encoder is to extract local contextual patterns for each residue and integrate them to form the global sequence feature of the protein. Subsequently, the original residue embedding and the global sequence feature extracted by the encoder are passed into a modified transformer decoder, as shown in Figure 1c. This decoder incorporates a cross‐attention mechanism and adapts the mask operation of the original transformer to ensure learning across the full length of the protein. The multi‐head cross‐attention mechanism enables ALLSites to capture interactions between each residue and other residues throughout the sequence. Finally, the embedding output by the decoder is fed into a classifier composed of fully connected layers (FCs), which predicts the probability of each residue being a binding site for different drug modalities. The ability of ALLSites to learn diverse residue features directly from protein sequence allows it to serve as a unified framework for identifying proteome‐wide binding sites across all drug modalities, including proteins, peptides, small molecules, carbohydrates, DNA, and RNA.

### 2.2 Evaluation of ALLSites in Identifying Binding Sites of Proteins/Peptides

Identifying PPI sites and PepPI sites is essential for the development of biologics such as protein‐ and peptide‐based therapeutics [43, 44]. To comprehensively evaluate the performance of ALLSites in identifying binding sites of proteins and peptides, we employed four commonly used benchmark datasets for PPI sites and two benchmark datasets for PepPI sites.

#### 2.2.1 Performance Evaluation of ALLSites in Predicting PPI Sites

We first compared the performance of ALLSites and 13 competing methods on the PPI‐Test70 dataset. The competing methods included four structure‐based methods (IntPred [45], SPPIDER [46], DeepPPISP [24], and EGRET [39]) and nine sequence‐based methods (ISIS [47], RF_PPI [48], PSIVER [49], SPRINGS [50], ProNA2020 [51], SCRIBER [52], DELPHI [25], DLPred [53], and EnsemPPIS [26]). Except for SPPIDER, ProNA2020, SCRIBER, and DLPred results, which were obtained using the web server, the results of the other methods were obtained by reproducing the source code or directly collected from the DeepPPISP literature [24], as they used the same PPI‐Train352 as the training dataset. As shown in Table 1, ALLSites consistently performed best in terms of AUROC, AUPRC, F1, and MCC metrics, surpassing all sequence‐based and structure‐based methods. Particularly, compared to the second‐best method, EnsemPPIS, ALLSites achieved improvements of 5.0% in AUROC and 8.1% in AUPRC. In terms of the F1 and MCC, ALLSites improved by 0.034 and 0.042, respectively. In another widely used PPI site prediction task (using PPI‐Train9982 as the training set and PPI‐Test355 as the test set), structural information was unavailable in the training data. Thus, the comparison was conducted exclusively among sequence‐based methods. As shown in Table 2, ALLSites still exhibited superior performance compared to all sequence‐based methods, achieving the highest scores in all metrics. ALLSites was 6.9% and 24.6% higher than EnsemPPIS in terms of AUROC and AUPRC, respectively, and was 0.081 and 0.096 higher in terms of F1 and MCC metrics, respectively.

**TABLE 1:** Performance evaluation of ALLSites on the PPI‐Test70 dataset. The best performance for each metric is highlighted in bold, and the second‐best performance is underlined.

Class	Method	ACC	AUROC	AUPRC	F1	MCC
Structure‐based	IntPred ^a^	0.672	−	−	0.332	0.165
	SPPIDER ^c^	0.667	0.518	0.235	0.273	0.063
	DeepPPISP ^b^	0.655	0.671	0.320	0.397	0.206
	EGRET ^b^	0.715	0.719	0.405	0.438	0.270
Sequence‐based	ISIS ^a^	0.622	−	0.240	0.267	0.097
	RF_PPI ^a^	0.598	−	0.210	0.258	0.118
	PSIVER ^a^	0.653	−	0.250	0.328	0.138
	SPRINGS ^b^	0.631	−	0.280	0.350	0.181
	ProNA2020 ^c^	**0.741**	−	−	0.258	0.106
	SCRIBER ^c^	0.616	0.635	0.307	0.370	0.159
	DELPHI ^b^	0.667	0.690	0.360	0.418	0.236
	DLPred ^c^	0.680	0.697	0.380	0.416	0.235
	EnsemPPIS ^b^	0.732	0.719	0.405	0.440	0.277
	ALLSites	0.720	**0.755**	**0.438**	**0.474**	**0.319**

^a^ Results reported by DeepPPISP.

^b^ Results generated by reproducing the source code.

^c^ Results obtained by using the web server. ProNA2020 only makes binary predictions, and its AUROC and AUPRC are not calculated.

**TABLE 2:** Performance evaluation of ALLSites on the PPI‐Test355 dataset. All the comparison methods use only protein sequences. The best performance for each metric is highlighted in bold, and the second‐best performance is underlined.

Method	ACC	AUROC	AUPRC	F1	MCC
SPRINGS ^a^	0.811	0.608	0.178	0.211	0.103
DLPred ^b^	0.835	0.724	0.272	0.308	0.214
SCRIBER ^b^	0.838	0.719	0.275	0.322	0.230
DELPHI ^a^	0.848	0.746	0.326	0.364	0.278
EnsemPPIS ^a^	0.821	0.770	0.354	0.385	0.291
ALLSites	**0.850**	**0.823**	**0.441**	**0.466**	**0.387**

^a^ Results generated by reproducing the source code.

^b^ Results obtained by using the web server.

**FIGURE 2:**
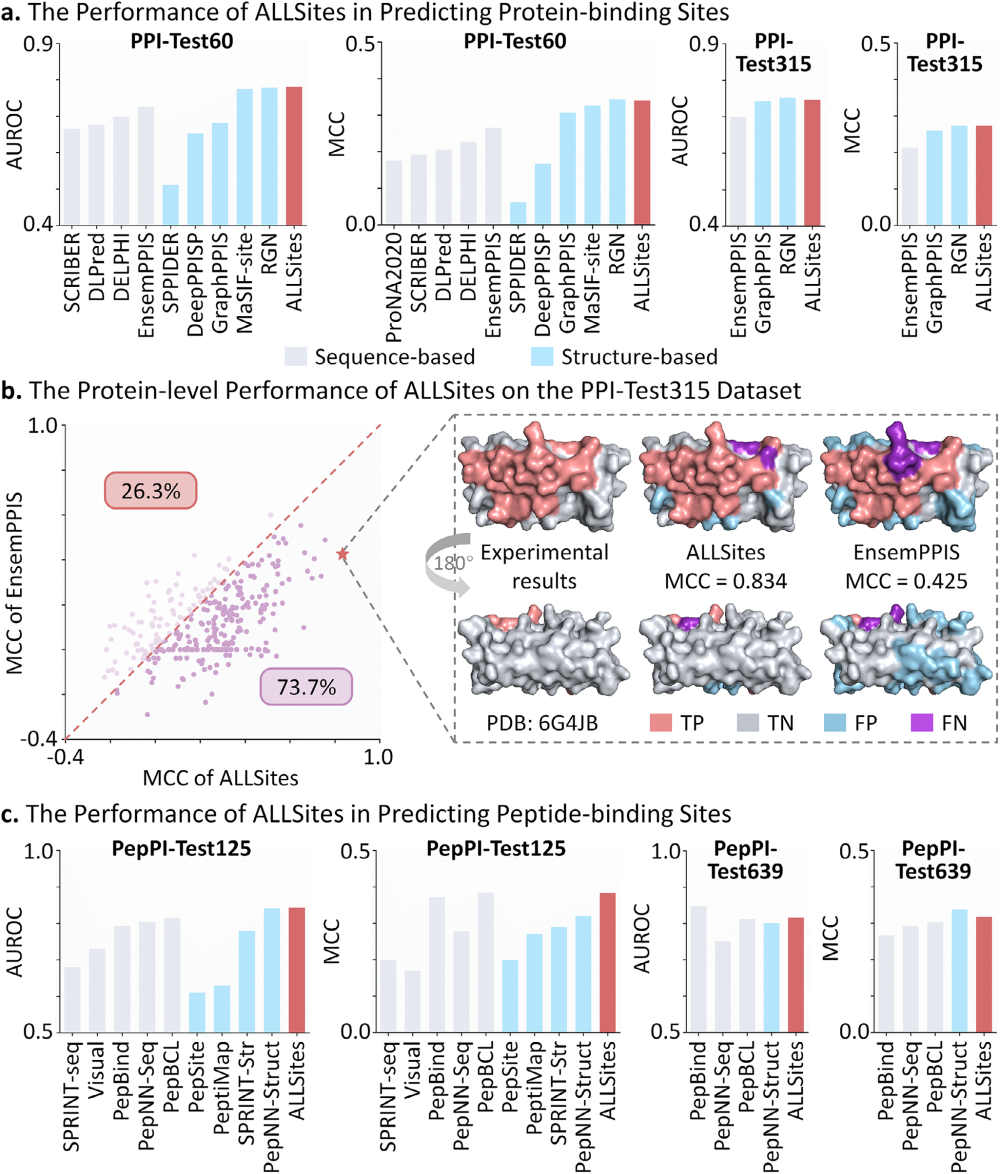
The performance of ALLSites in identifying binding sites of proteins and peptides. a). The performance of ALLSites in predicting protein‐binding sites. ALLSites is evaluated on the PPI‐Test60 and PPI‐Test315 datasets in terms of AUROC and MCC. Sequence‐based methods are indicated by gray bars, and structure‐based methods are indicated by blue bars. b). The protein‐level performance of ALLSites on the PPI‐Test315 dataset. The MCC metric is calculated for each protein based on ALLSites’ predictions. A specific protein (PDB ID: 6G4JB) is presented to illustrate the predictions of ALLSites and EnsemPPIS alongside the corresponding experimental results. c). The performance of ALLSites in predicting peptide‐binding sites. ALLSites is evaluated on the PepPI‐Test125 and PepPI‐Test639 datasets in terms of AUROC and MCC. Source data are provided in Tables S2 to S5.

In addition, ALLSites was evaluated on the PPI‐Test60 and PPI‐Test315 datasets. The model evaluated on these two test sets was the same model, which was trained on the PPI‐Train335 dataset using the same training scheme. As presented in Figure 2a, in terms of the key metrics AUROC and MCC, ALLSites consistently outperformed all sequence‐based methods by a large margin and achieved performance comparable to the best structure‐based method, RGN [54]. By contrast, the second‐best sequence‐based method, EnsemPPIS, consistently underperformed compared to the structure‐based methods GraphPPIS and RGN in terms of MCC. It was worth noting that, on the PPI‐Test60 dataset, ALLSites showed slightly better performance than MaSIF‐site [55], which used advanced geometric deep learning to learn surface features from protein structures. We also performed a calibration analysis of ALLSites on these two test datasets. As illustrated in Figure S1, the reliability diagram demonstrated well‐calibrated predictions. Specifically, ALLSites achieved Brier scores of 0.153 and 0.163 on PPI‐Test60 and PPI‐Test315, respectively. Both Brier scores were below the commonly accepted threshold of 0.25, suggesting that their predicted probabilities were reasonably reliable [56].

In fact, the protein representation module in ALLSites could leverage newer PLMs beyond ESM‐2. A notable example is ESM‐C, a parallel model family specifically designed to generate biologically meaningful protein representations alongside the ESM3 generative models [57]. Therefore, it is necessary to compare the performance of ALLSites when using either ESM‐2 (ALLSites‐ESM2) or ESM‐C (ALLSites‐ESMC) for protein representation. Due to hardware constraints, we selected ESM‐C 600M to represent protein sequences and evaluated ALLSites on PPI‐Test70, PPI‐Test315, and PPI‐Test60. It generated a 1,152‐dimensional embedding for each protein residue. As shown in Table S1, replacing ESM‐2 with ESM‐C 600M led to a slight improvement in AUROC and AUPRC on PPI‐Test70, but resulted in decreased performance across other metrics, including ACC, F1, and MCC. Notably, on both PPI‐Test60 and PPI‐Test315, the use of ESM‐C caused a consistent and substantial drop in nearly all evaluation metrics, particularly MCC, which decreased by 0.037 and 0.032, respectively. These findings indicated that, for the task of PPI site prediction, adopting the newer ESM‐C did not further enhance the predictive performance of ALLSites. This conclusion aligned with a recent large‐scale benchmarking study of PLMs [58], reinforcing that the choice of ESM‐2 as the protein representation backbone in ALLSites was well justified.

Furthermore, we additionally assessed the protein‐level performance of ALLSites on the PPI‐Test315 and PPI‐Test60 datasets. As shown in Figure 2b, compared with the current best sequence‐based method EnsemPPIS, ALLSites achieved higher MCC scores on 73.7% of the 315 proteins in the PPI‐Test315 dataset. For a selected case protein (PDB ID: 6G4JB), ALLSites produced fewer false positives and false negatives, resulting in an MCC of 0.834, whereas EnsemPPIS achieved an MCC of only 0.425. Similar results were observed on the PPI‐Test60 dataset. As shown in Figure S2a, ALLSites outperformed EnsemPPIS in terms of MCC on 81.7% of the proteins. For the case protein (PDB ID: 4EMJB), EnsemPPIS predicted a large number of false positive PPI sites, leading to an MCC less than half of that achieved by ALLSites. However, in the PPI‐Test60 test set, ALLSites still yielded lower MCC values than EnsemPPIS for 18.3% of the proteins. Taking a case protein as an example (PDB ID: 4HLUA), the MCC value of ALLSites’ prediction was only 0.095, compared to 0.333 for EnsemPPIS. Although ALLSites produced fewer false‐positive PPI sites, its extremely low number of true‐positive predictions resulted in a substantially lower MCC value.

In terms of the AUROC metric, ALLSites also achieved higher AUROC values than EnsemPPIS on the majority of proteins, as shown in Figure S2b. In summary, these results indicated that ALLSites achieved SOTA predictive performance in PPI site prediction at both the residue and protein levels.

#### 2.2.2 Performance Evaluation of ALLSites in Predicting PepPI Sites

The performance evaluation of ALLSites in PepPI site prediction was conducted on two tasks. The first task involved training on PepPI‐Train1154 and testing on PepPI‐Test125, while the second task used PepPI‐Train640 for training and PepPI‐Test639 for testing. A total of nine PepPI site prediction methods were compared with ALLSites, including five sequence‐based methods (SPRINT‐seq [59], Visual [60], PepBind [27], PepNN‐Seq [61], and PepBCL [42]) and four structure‐based methods (PepSite [28], PeptiMap [62], SPRINT‐Str [63], and PepNN‐Struct [61]). Since the same training dataset and model training strategy were employed, the performance metrics of competing methods were obtained from the PepBCL literature [42].

As shown in Figure 2c, ALLSites exhibited excellent predictive performance in both tasks. Specifically, on the PepPI‐Test125 dataset, ALLSites outperformed all sequence‐based (gray bars) and structure‐based (blue bars) methods in both AUROC and MCC metrics. In terms of AUROC, ALLSites surpassed all sequence‐based methods and slightly exceeded the best structure‐based method, PepNN‐Struct. Regarding the MCC metric, ALLSites outperformed all structure‐based methods, with only a 0.002 margin below the best‐performing method, PepBCL. On PepPI‐Test639, PepBind and PepNN‐Struct achieved the highest scores in AUROC and MCC, respectively, while ALLSites consistently ranked second, highlighting its strong robustness. These findings demonstrated that ALLSites was capable of accurately identifying PepPI sites on proteins, offering a valuable tool for advancing peptide drug discovery and design.

### 2.3 Evaluation of ALLSites in Identifying Binding Sites of Small Molecules/Carbohydrates

#### 2.3.1 Performance Evaluation of ALLSites in Predicting SMPI Sites

**FIGURE 3:**
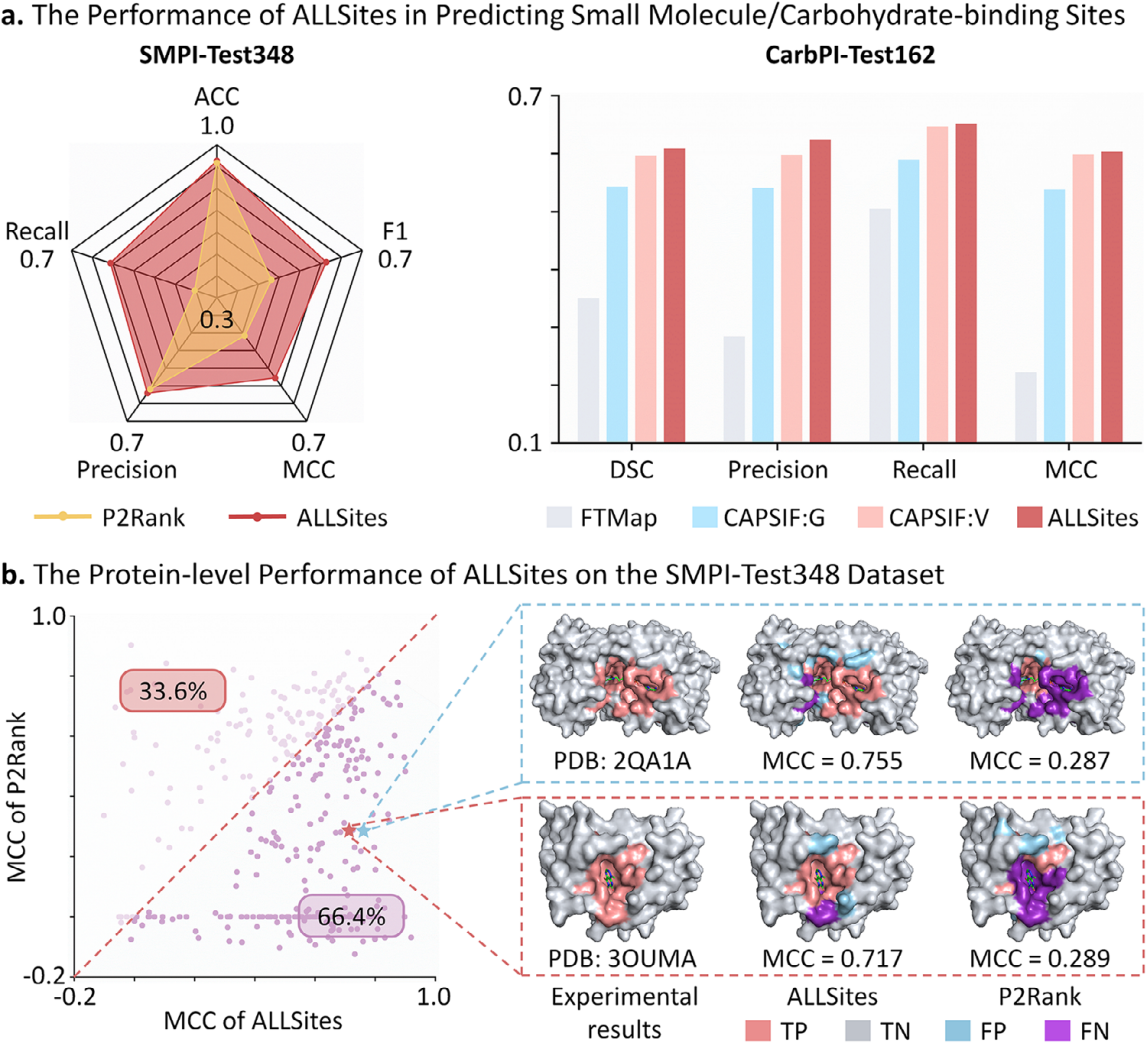
The performance of ALLSites in identifying binding sites of small molecules and carbohydrates. a). The residue‐level performance of ALLSites in predicting SMPI sites and CarbPI sites. ALLSites is evaluated on the SMPI‐Test348 and CarbPI‐Test162 datasets. All the compared methods are based on structural information. Source data are provided in Tables S6 and S7. b). The protein‐level performance of ALLSites on the SMPI‐Test348 dataset. The MCC metric is calculated for each protein based on ALLSites’ predictions. Two specific proteins (PDB ID: 2QA1A and PDB ID: 3OUMA) are presented to illustrate the predictions of ALLSites and P2Rank alongside the corresponding experimental results.

Small molecules represent the most prevalent molecular modality among approved therapeutics, making the identification of potential SMPI sites on proteins crucial for the development of novel small‐molecule drugs [64]. To evaluate the performance of ALLSites in predicting SMPI sites, a new benchmark dataset of SMPI sites was constructed based on the sc‐PDB database [65], which comprised 2 324 non‐redundant proteins with low pairwise sequence identity. These proteins were randomly partitioned into a training set (SMPI‐Train1628), a validation set (SMPI‐Valid348), and a test set (SMPI‐Test348). We compared the performance of ALLSites and P2Rank [29]. (a widely used structure‐based method) on the SMPI‐Test348 dataset. Following the instructions described in the original publication, the performance of P2Rank was calculated using the top‐ranked predicted pocket. It should be noted that P2Rank's predictive performance was obtained by directly loading its pre‐trained model parameters for inference. Consequently, the comparison with ALLSites may be inherently unfair due to potential differences in the training data used. Nevertheless, from a practical application standpoint, evaluating ALLSites against the pre‐trained P2Rank remains meaningful. As illustrated in the left panel of Figure 3a, ALLSites outperformed P2Rank across all five metrics, including accuracy, recall, precision, F1, and MCC. Particularly, ALLSites achieved F1, MCC, and recall scores of 0.601, 0.560, and 0.593, respectively, achieving improvements of 0.151, 0.136, and 0.232 over P2Rank.

Similarly, we assessed the protein‐level performance of ALLSites on the SMPI‐Test348 dataset. As illustrated in Figure 3b, among the 348 unique proteins, ALLSites achieved higher MCC values than P2Rank for 66.4% of the cases. As demonstrated by two representative proteins (PDB ID: 2QA1A and 3OUMA), ALLSites achieved MCC scores of 0.755 and 0.717, markedly surpassing those of P2Rank. Obviously, the binding sites predicted by ALLSites were much closer to the true binding pockets, whereas P2Rank exhibited a substantially higher number of false negative predictions (purple residues). Notably, ALLSites achieved superior performance compared to the well‐established P2Rank algorithm without using any structural information, highlighting its powerful capability in extracting binding site features from sequence alone.

#### 2.3.2 Performance Evaluation of ALLSites in Predicting CarbPI Sites

Research on the interactions between carbohydrates and proteins has already led to several approved drugs [66, 67, 68]. A considerable portion of carbohydrate molecules falls within the category of small molecules. However, the distinctive chemical properties of carbohydrates, especially their rich hydroxyl groups, generate binding sites that are fundamentally different from those of typical small molecules [69, 70]. Therefore, it is essential to evaluate the performance of ALLSites in identifying CarbPI sites.

The benchmark dataset used to assess ALLSites’ ability in predicting CarbPI sites was derived from a previous study and consisted of a training set (CarbPI‐Train517), a validation set (CarbPI‐Valid129), and a test set (CarbPI‐Test162) [30]. Three structure‐based methods were employed for comparison with ALLSites, including one general small‐molecule binding site prediction tool (FTMap [71]) and two carbohydrate‐specific binding site prediction tools (CAPSIF: V^30^ and CAPSIF: G^30^). Since the same training and evaluation protocols were applied, the performance metrics of these competing methods were directly obtained from the original literature [30]. To ensure consistency and comparability, we adopted the same evaluation metrics as the original study, calculating the average metric values across all proteins in the test set. As shown in the right panel of Figure 3a, ALLSites outperformed all three methods in terms of average DICE, precision, recall, and MCC. The performance of ALLSites was substantially higher than that of FTMap, with average DICE and MCC values improved by 0.258 and 0.381, respectively. The inferior performance of FTMap can be attributed to its design for general small‐molecule pocket prediction, as it is not specifically tailored for identifying CarbPI sites. These results further confirm the fact that carbohydrate‐binding sites are distinctly different from conventional small‐molecule binding sites. Moreover, ALLSites is highly portable and can be adapted to other small‐molecule drug modalities, such as the prediction of covalent binding sites [72].

### 2.4 Evaluation of ALLSites in Identifying Binding Sites of Nucleic Acids

#### 2.4.1 Performance Evaluation of ALLSites in Predicting DPI and RPI Sites

Nucleic acid‐protein interactions play crucial roles in numerous essential cellular processes, such as DNA replication, transcription, and translation [73, 74]. Elucidating the molecular mechanisms underlying these interactions, including the characterization of DNA‐ and RNA‐binding sites on proteins, can facilitate the development of drugs to treat diseases caused by aberrant regulation between proteins and nucleic acids [75, 76, 77]. To assess ALLSites’ ability in identifying nucleic acid binding sites, we utilized two benchmark datasets for DPI sites and one for RPI sites.

**FIGURE 4:**
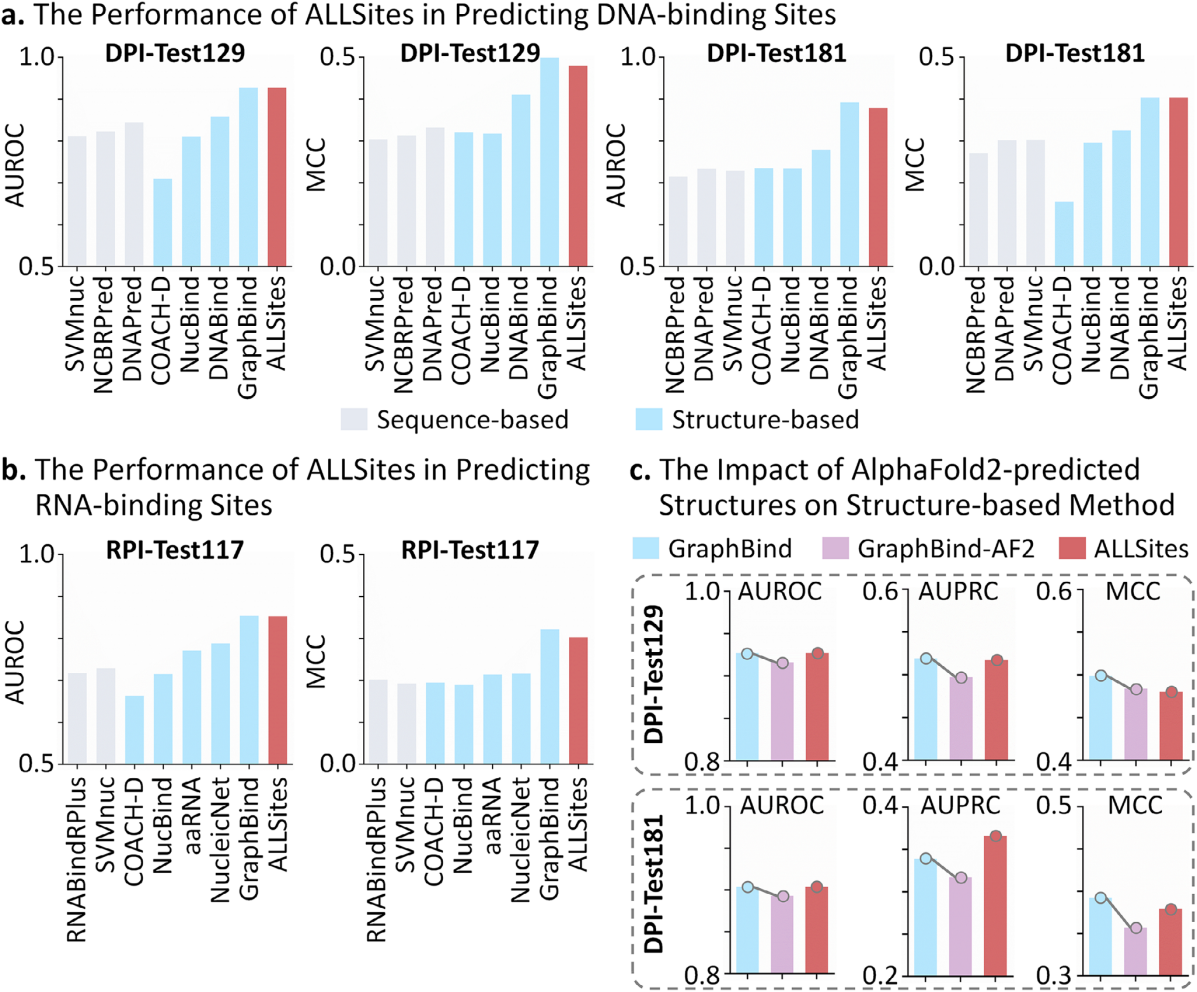
The performance of ALLSites in identifying binding sites of nucleic acids. a). The performance of ALLSites in predicting DNA‐binding sites. ALLSites is evaluated on the DPI‐Test129 and DPI‐Test181 datasets in terms of AUROC and MCC. Sequence‐based methods are indicated by gray bars, and structure‐based methods are indicated by blue bars. b). The performance of ALLSites in predicting RNA‐binding sites. ALLSites is evaluated on the RPI‐Test117 dataset in terms of AUROC and MCC. c). The impact of AlphaFold2‐predicted structures on the structure‐based method. The impact of predicted structures on GraphBind's performance was evaluated using the DPI‐Test129 and DPI‐Test181 datasets. Blue and purple bars represent the performance of GraphBind using experimentally determined structures and predicted structures (GraphBind‐AF2), respectively. Source data are provided in Tables S8 to S14.

In the task for DPI site prediction, three sequence‐based methods (SVMnuc [78], NCBRPred [79], and DNAPred [32]) and four structure‐based methods (COACH‐D [80], NucBind [78], DNABind [33], and GraphBind [41]) were selected for performance comparison with ALLSites. The results for GraphBind were obtained by reproducing the source code, while the performances of the other methods were retrieved using their respective web servers. A single model trained on the DPI‐Train573 dataset was applied to evaluate prediction performance on both the DPI‐Test129 and DPI‐Test181 test sets. As shown in Figure 4a, ALLSites significantly outperformed all sequence‐based methods and the majority of structure‐based methods on both AUROC and MCC metrics across the two test sets. Compared to the best‐performing sequence‐based method, ALLSites achieved improvements of 9.7% and 12.6% in AUROC on DPI‐Test129 and DPI‐Test181, respectively, along with MCC improvements of 0.148 and 0.150. Although ALLSites showed slightly lower MCC scores than the best‐performing structure‐based method, GraphBind, it achieved comparable AUROC performance on both test sets.

In the benchmarking task for RPI site prediction, the performance of ALLSites was compared with two sequence‐based methods (RNABindRPlus [81] and SVMnuc [78]) and five structure‐based methods (COACH‐D [80], NucBind [78], aaRNA [82], NucleicNet [34], and GraphBind [41]). The results for NucleicNet and GraphBind were obtained by reproducing the provided source code, while the performance of the other methods was obtained using their web servers. As shown in Figure 4b, consistent with the results observed in the DPI site prediction, ALLSites outperformed all sequence‐based methods and the majority of structure‐based approaches. Specifically, ALLSites ranked second in both AUROC and MCC metrics, with AUROC values comparable to those of the best‐performing structure‐based method, GraphBind. These findings provide strong evidence that ALLSites can accurately identify nucleic acid binding sites solely from protein sequences.

#### 2.4.2 Performance Evaluation of Structure‐Based Method Using Predicted Structures

Although ALLSites consistently shows slightly lower performance than the best‐performing structure‐based method, GraphBind, in the tasks for both DPI and RPI site prediction, it offers broader applicability across the proteome due to its reliance solely on sequence information. This advantage is significant because structure‐based methods are limited by the low availability of high‐resolution structures and their high sensitivity to structural errors. Specifically, only about 35% of human proteins have experimentally determined crystal structures, and in many cases, these structures cover only a fragment of the full sequence [35]. Moreover, although advanced protein structure prediction tools (such as AlphaFold2 [83] and RoseTTaFold [84]) can partially alleviate the scarcity of structural data, the inherent deviations between predicted and native structures often substantially degrade the performance of structure‐based prediction methods.

To elucidate the limitations of structure‐based methods, we evaluated the impact of AlphaFold2‐predicted structures on GraphBind using the DPI‐Test129 and DPI‐Test181 datasets. As shown in Figure 4c, GraphBind exhibited a clear performance decline across all three metrics (AUROC, AUPRC, and MCC) when predicted structures were used instead of experimentally resolved ones. On the DPI‐Test129 dataset, GraphBind's AUROC and AUPRC dropped below those of ALLSites, with MCC values becoming comparable to ALLSites. On DPI‐Test181, the use of predicted structures led to GraphBind underperforming ALLSites across all three metrics. These results confirm that structure‐based methods are highly sensitive to structural errors, and even state‐of‐the‐art structure prediction tools cannot fully compensate for the limitations imposed by the scarcity of experimentally resolved structures. Source data for all benchmark results were provided in Tables S2–S14.

ALLSites exhibits a very fast inference speed. On a single NVIDIA V100 GPU, it can screen the entire human proteome (comprising 20 420 reviewed human proteins from the UniProt database) within 16 h. On average, it takes 2.81 s per protein and only 0.0075 s per residue. In conclusion, given its reliance solely on protein sequence and its fast inference speed, ALLSites is well‐suited for proteome‐wide mapping of binding sites across all drug modalities.

## 3 Materials and Methods

### 3.1 Dataset Collection and Data Processing

#### 3.1.1 Benchmark Datasets for PPI Site and PepPI Site Prediction

In this study, four commonly used benchmark datasets for PPI site prediction and two benchmark datasets for PepPI site prediction were used to assess the performance of ALLSites in identifying binding sites of proteins and peptides.

The first PPI site benchmark (PPI‐Train352 and PPI‐Test70) was sourced from DeepPPISP [24], originally curated from the PDB [85] through a six‐step data filtering process [49]. Both datasets comprised proteins with less than 25% sequence homology, ensuring low redundancy in model training and evaluation. A surface residue was annotated as a PPI site if its absolute solvent accessibility decreased by at least 1.0 Å^2^ upon protein binding [86]. A subset of 50 proteins was randomly selected from PPI‐Train352 to form a hold‐out validation set. The second PPI site benchmark (PPI‐Train9982 and PPI‐Test355) was collected by DELPHI [25]. The PPI‐Test355 dataset was built based on the BioLip database [87] and comprised 355 non‐redundant proteins with pairwise sequence similarity below 25%. Residues were annotated as PPI sites if the distance between any two atoms from different chains was less than 0.5 Å plus the sum of their van der Waals radii. The PPI‐Train9982 dataset was collected from a previous study [88], with all proteins exhibiting less than 25% sequence similarity to those in the PPI‐Test355 set. A total of 1 110 sequences were randomly selected from PPI‐Train9982 to form a validation set, while the remaining sequences were used for model training. The PPI‐Train9982 dataset lacks structural annotations and is therefore unsuitable for training structure‐based PPI site prediction methods. The third PPI site benchmark (PPI‐Train335 and PPI‐Test60) was constructed by GraphPPIS [36]. The sequences in both datasets also exhibited less than 25% sequence similarity. To ensure a fair comparison, the PPI‐Train335 and PPI‐Test60 datasets were identical to those employed in the GraphPPIS study. Furthermore, the model trained on PPI‐Train335 was also evaluated on the PPI‐Test315 dataset [36], which was a previously published dataset comprising proteins with less than 25% sequence identity with those in PPI‐Train335.

The two PepPI benchmark datasets were directly adopted from a previous study [42]. The first benchmark (PepPI‐Train1154 and PepPI‐Test125) was originally introduced in the SPRINT‐Str study [63]. The second benchmark (PepPI‐Train640 and PepPI‐Test639) was derived from a previous work as well [27]. Both benchmarks underwent a similar preprocessing pipeline, and sequence identity between the training and test sets was reduced to a maximum of 30% using the BLASTClust in the BLAST package to ensure reliable performance evaluation [89].

#### 3.1.2 Benchmark Datasets for SMPI Site and CarbPI Site Prediction

In this study, to evaluate the performance of ALLSites in identifying small‐molecule binding sites, we constructed a new benchmark dataset for SMPI site prediction. First, 17 594 small‐molecule‐protein complex structures were downloaded from the sc‐PDB database [65]. According to the sc‐PDB definition, a protein residue was considered as a binding site if any of its atoms was located within 6.5 Å of a ligand atom. Following the protocol adopted in a previous study [90], binding site annotations from multiple PDB entries of the same protein were mapped to their corresponding UniProt sequences, resulting in 4,993 unique protein sequences. To avoid bias in performance evaluation, the sequence identity was reduced to 30% using the BLASTClust algorithm. This yielded a non‐redundant set of 2 324 proteins. From this set, 348 proteins were randomly selected to form the independent test dataset (SMPI‐Test348), while the remaining 1 976 proteins constituted the training set. Furthermore, an additional 348 proteins were randomly selected from SMPI‐Train1976 to form a validation set (SMPI‐Valid348), leaving 1 628 proteins as the final training set (SMPI‐Train1628) for model training.

The benchmark dataset for CarbPI site prediction was obtained from the CAPSIF study [30]. It comprises 517 proteins for training (Carb‐Train517), 129 for validation (Carb‐Valid129), and 162 for an independent test (Carb‐Test162). All protein structures in this benchmark had a resolution lower than 3.0 Å, and sequence identity between any two proteins was below 30%. A residue was defined as a CarbPI site if any of its heavy atoms were within 4.2 Å of a heavy atom in the bound carbohydrate.

#### 3.1.3 Benchmark Datasets for DPI Site and RPI Site Prediction

Two benchmark datasets for DPI site prediction and one benchmark dataset for RPI site were collected to evaluate the performance of ALLSites in identifying binding sites of nucleic acids.

The first benchmark dataset for DPI site prediction (DPI‐Train573 and DPI‐Test129) was adopted from the GraphBind study [41]. The DNA‐binding proteins were initially curated from the BioLip database and processed through a series of filtering steps [87], resulting in 573 proteins in the training set (DPI‐Train573) and 129 in the independent test set (DPI‐Test129). Sequence identity between proteins in the training and test sets was below 30%. A residue was defined as a DPI site if the shortest atomic distance between it and the DNA molecule was less than 0.5 Å plus the sum of the van der Waals radii of the two closest atoms. Another independent test dataset for DPI site prediction, DPI‐Test181, was collected from a previous study [91]. The proteins in DPI‐Test181 shared less than 30% sequence identity with those in the DPI‐Train573 training set, enabling an unbiased evaluation. Therefore, the model trained on DPI‐Train573 was also assessed on DPI‐Test181 to validate its generalizability.

The benchmark dataset for RPI site prediction (RPI‐Train495 and RPI‐Test117) was also obtained from the GraphBind study [41]. The data preprocessing pipeline and binding site definition criteria were kept consistent with those applied to the DPI‐Train573 and DPI‐Test129 datasets. Sequence identity between proteins in RPI‐Train495 (495 proteins) and RPI‐Test117 (117 proteins) was also below 30%, ensuring minimal sequence redundancy and a reliable evaluation.

Notably, class imbalance is prevalent across all benchmark datasets, where the number of non‐binding sites exceeds that of binding sites. Dataset statistics for all benchmark tasks are summarized in Table S15, which includes protein counts, numbers of binding and non‐binding residues, and the fraction of binding residues among all residues.

### 3.2 Protein Representation

Protein sequences were represented using ESM‐2, a transformer‐based protein language model pre‐trained on 65 million protein sequences with 3B parameters [92]. ESM‐2 leverages large‐scale self‐supervised pre‐training to extract semantic knowledge at a molecular level, enabling the inference of deep embeddings that align with biological semantics. This language model was selected for its ability to capture evolutionary information and complex structural patterns within protein sequences without requiring explicit structural data. For each protein sequence, residue‐level features were extracted using ESM‐2, with each amino acid represented as a fixed‐dimensional vector of 2 560 features. This provided a rich, context‐aware representation for every residue position, which was crucial for accurate binding site prediction.

### 3.3 Model Architecture of ALLSites

ALLSites is a novel deep learning framework for protein binding site prediction, featuring an encoder‐decoder architecture enhanced with a cross‐attention mechanism. As illustrated in Figure 1a, its framework comprises three key components: (1) a protein feature encoder, (2) a cross‐attention decoder, and (3) a classification module. By capturing both local and global protein features, as well as residue interaction features, ALLSites can identify potential binding sites across all drug modalities.

#### 3.3.1 Protein Feature Encoder

The protein feature encoder extracts meaningful representations from protein sequences. The structure of the encoder is depicted in Figure 1b. Initially, the encoder maps the input protein features through a fully connected layer to obtain a hidden representation of dimension *d_hid_*. The mapped features are then processed through a series of 1D convolutional layers (Conv1D) with gated linear units (GLU) activation functions [93]. Specifically, the encoder contains *n* convolutional layers, each with kernel size *k* and padding (*k* − 1)/2 to maintain the sequence length. Each convolutional layer produces an output of dimension 2 × *d_hid_*, which is then processed through the GLU activation function to obtain an output of dimension *d_hid_*.

To facilitate gradient flow through the network, we employed residual connections that combined the input and output of each convolutional layer with a scaling factor. Layer normalization is applied to the final output to stabilize the training process. The protein encoder's computation is defined by Equation (1).

(1) $$H_{p}=\mathrm{Encoder}\left(P\right)$$

where $P\in R^{B\times L_{p}\times d_{p}}$ $P\in R^{B\times L_{p}\times d_{p}}$ represents the protein features with batch size *B*, sequence length *L_p_*, and input feature dimension *d_p_*, and $H_{p}\in R^{B\times L_{p}\times d_{hid}}$ is the encoded protein representation.

#### 3.3.2 Cross‐Attention Decoder

The decoder processes the encoded global protein features while also attending to the residue interactions. As shown in Figure 1c, the decoder architecture comprises multiple decoder layers, each featuring a cross‐attention mechanism and a position‐wise feed‐forward network, both enhanced by residual connections and layer normalization [94]. In the decoder layer, the local protein features first undergo self‐attention to capture internal relationships within the local sequence. The cross‐attention mechanism enables the model to focus on residue interactions potentially involved in binding interactions. Subsequently, the position‐wise feed‐forward network, comprising two convolutional layers with a ReLU activation in between, enhances the model's representational capacity.

The cross‐attention mechanism is a critical component that enables the model to capture long‐range dependencies between the potential binding sites and other protein residues. Our implementation follows the multi‐head attention paradigm, where the attention is computed in parallel across multiple representation subspaces. For each attention head, we compute query (***Q***), key (***K***), and value (***V***) from the input features. As shown in Equation (2), the attention scores are calculated as scaled dot products between query and keys, followed by softmax normalization.

(2) $$\mathrm{Attention}\left(Q,K,V\right)=\mathrm{softmax}\left(\frac{QK^{T}}{\sqrt{d_{k}}}\right)V$$

where *d_k_* is the dimensionality of the key. The outputs from all attention heads are concatenated and projected to obtain the final attention output. The decoder can be expressed as Equation (3).

(3) $$H_{l},A=\mathrm{Decoder}\left(L,H_{p},M_{l},M_{p}\right)$$

where $L\in R^{B\times L_{l}\times d_{l}}$ represents the local features of a specific residue, ***M**_l_* and ***M**_p_* are the mask matrices for local and global protein features, respectively, $H_{l}\in R^{B\times d_{hid}}$ is the processed local interaction representation, and ***A*** represents the attention weights.

#### 3.3.3 Classification Module

After obtaining the processed local interaction representation, we employ a significance‐weighted aggregation strategy to combine information from all local features. The norm of each local feature representation is used to compute a softmax‐normalized significance score, which is then used to weight its contribution to the final representation. ***H***_agg_. The computation formula is shown in Equation (4).

(4) $$H_{\mathrm{agg}}=\sum_{j=1}^{L_{l}}\frac{\exp(|\left|H_{l}^{j}\right|{|}_{2})}{\sum_{k=1}^{L_{l}}\exp\left(|\left|H_{l}^{k}\right|{|}_{2}\right)}\cdot H_{l}^{j}$$

The aggregated representation is passed through a series of FCs with the ReLU activation to predict the binding probability.

(5) $$z=FC_{3}\left(FC_{2}\left(FC_{1}\left(H_{\mathrm{agg}}\right)\right)\right)$$

where $z\in R^{B\times 2}$ is the logits for binary classification of binding site or non‐binding site.

### 3.4 Model Training and Implementation

#### 3.4.1 Training Procedure

ALLSites was trained on the binding site classification task using the weighted cross‐entropy loss function. Since the number of binding residues in all prediction tasks was substantially greater than that of non‐binding residues, assigning a higher loss weight to binding residues could enhance the model's ability to predict them accurately. In this study, the loss weight for each task was treated as a hyperparameter and optimized accordingly. The final loss weight values used for all tasks were provided in Table S15. The weight loss was set prior to model training and remained fixed throughout the training process. The computation formula of weighted cross‐entropy loss was shown in Equation (6).

(6) $$\mathrm{Loss}=-\sum_{i=1}^{N}w*y_{i}\log\hat{y_{i}}+\left(1-y_{i}\right)\log\left(1-\hat{y_{i}}\right)$$

where *y_i_*  is the true label of residue *i*, $\hat{y_{i}}$ is the predicted probability of residue *i* being a binding site, and *N* is the total number of all residues, *w* is the loss weight of a true binding site.

To enhance training stability and convergence, we employed the RAdam optimizer combined with the Lookahead optimization technique [95]. The regularization methods, including dropout and weight decay, were applied to improve the capacity of generalization. For each drug modality's binding site, ALLSites was trained separately using the corresponding binding site dataset. In each prediction task, unless otherwise specified, ALLSites adopted the same training scheme as the competing methods.

Specifically, for PPI‐Test70, PPI‐Test355, SMPI‐Test348, and CarbPI‐Test162, models were trained on their respective training sets, evaluated on the corresponding validation sets for hyperparameter tuning, and the best‐performing models were finally evaluated on the test sets. For PPI‐Test60 and PPI‐Test315, the five‐fold cross‐validation was first performed on the PPI‐Train335 dataset to determine the best hyperparameters. Finally, the model was retrained on the entire PPI‐Train335 dataset using these best hyperparameters, and the model performance was evaluated on PPI‐Test60 and PPI‐Test315, respectively. The model training schemes for the two PepPI site prediction tasks were consistent with the original studies from which the datasets were sourced. For PepPI‐Train1154, the ten‐fold cross‐validation was conducted to identify the best hyperparameters. Subsequently, the model was retrained on the entire PepPI‐Train1154 dataset using the best hyperparameters and evaluated on the PepPI‐Test125 dataset. For PepPI‐Train640, a random subset of 128 proteins was first held out from the training set to form a validation set, used for hyperparameter selection. The performance of the best‐performing model was then evaluated on the PepPI‐Test639 test set. For DPI‐Train573 and RPI‐Train495, following the training scheme described in GraphBind [41], the training sets were randomly split into training and validation subsets in an 8:2 ratio. This splitting and training process was repeated ten times to generate ten independent models. The performance of each model was evaluated on its respective test set (DPI‐Test129, DPI‐Test181, or RPI‐Test117). The reported performance of ALLSites on these test sets represented the average performance across ten models. An early stopping strategy with a patience of ten epochs was employed in all tasks to mitigate overfitting.

#### 3.4.2 Model Implementation

ALLSites was configured with a series of settings. The hidden dimension (*d_hid_*) was 128; the number of encoder layers was 3; the number of decoder layers was 3; the number of attention heads was 8; the hidden dimension in the position‐wise feed‐forward network was 256; the kernel size of Conv1D was 7; and the weight decay was set to 1E‐4. Additionally, the four most influential hyperparameters (including batch size, learning rate, dropout rate, and loss weight) were optimized based on the model's predictive performance on the validation dataset. Given the large dataset size arising from treating each residue as an individual sample, ALLSites supported distributed training to accelerate model training. ALLSites was implemented in Python 3.10 and Pytorch 1.12.0 (http://pytorch.org/). All models were developed on the platform with Intel(R) Xeon(R) Gold 6132 CPU @ 2.60GHz, NVIDIA(R) Tesla(R) V100 32GB GPU, and 263GB RAM on CentOS Linux release 7.9.2009 (Core).

### 3.5 Evaluation Metrics

Several evaluation metrics were used to evaluate the model performance, including accuracy (ACC), precision, recall, area under the receiver operating characteristic curve (AUROC), area under the precision‐recall curve (AUPRC), F1 score, and Matthews correlation coefficient (MCC). Due to the class imbalance inherent in binding site datasets, the MCC metric is a particularly important metric, as it provides a robust assessment that accounts for imbalance in the data [96]. For the two test sets, PPI‐Test60 and PPI‐Test315, we further performed a calibration analysis of ALLSites based on the Brier score. It has been reported that the Brier score ranges from 0 to 1, and a Brier score below 0.25 indicates that the model's predictions are reliable [56]. Additionally, the Dice similarity coefficient (DSC) was calculated for the CarbPI site prediction task [97]. Unlike the other tasks, performance evaluation for CarbPI involved calculating metrics for each individual protein and then averaging these results across the entire test set. All the metrics were calculated using Scikit‐learn [98]. The formulas for computing these evaluation metrics were provided in Supporting Information

Table S16 explicitly documents the sources of the evaluation performance for all baseline methods across the assessed tasks. In general, for those methods whose results were obtained by reproducing their source code, we employed the same training and evaluation data splits as well as identical training protocols used by ALLSites. For certain methods, results were retrieved from their respective web servers primarily because their source code was not publicly available for retraining. For other methods, reported performance values were directly extracted from the original literature, as these studies utilized the same training data and training schemes as ALLSites, thereby ensuring a fair comparison.

### 3.6 Statistical Analysis

In this work, the benchmark results of ALLSites on DPI‐Test129, DPI‐Test181, and RPI‐Test117 datasets were obtained from ten independent runs. The results were presented as mean ± standard deviation (SD).

## Author Contributions

F.Z., H.B.D., F.C.L., and M.J.M. conceived the idea, designed the research, and wrote the manuscript. M.J.M., M.K.L. and Z.M.Z. constructed the model and performed benchmark evaluation. Y.L.R., X.Y.Y., Z.Q.P. and Y.Z. performed data analysis. H.Y., L.Y.Z., S.K.G. and Y.Z. collected the data. All authors have approved the latest version of the manuscript.

## Conflicts of Interest

The authors declare no conflicts of interest.

## Supporting information




**Supporting File**: advs73503‐sup‐0001‐SuppMat.docx.

## Data Availability

All benchmark datasets are available on GitHub (https://github.com/idrblab/ALLSites/). Source data for benchmark evaluation results are fully provided in the Supplementary Information.
