# Supplementary material for: Accurate Identification of Protein Binding Sites for All Drug Modalities Using ALLSites
Source: Adv Sci (Weinh). 2025 Dec 27;13(10):e16530. doi: 10.1002/advs.202516530 (PMC12915145; doi:10.1002/advs.202516530)
Supplement: Supplementary file 1 — Supporting File: advs73503‐sup‐0001‐SuppMat.docx. [file ADVS-13-e16530-s001.docx]

***Supplementary Information for:***

**Accurate Identification of Protein Binding Sites for All Drug Modalities Using ALLSites**

Minjie Mou**^1^**^,^**^2^**^,^†, Mingkun Lu**^2^**^,^†, Zhimeng Zhou**^2^**^,^†, Yanlin Ren**^2^**, Xinyuan Yu**^2^**, Ziqi Pan**^2^**, Yuan Zhou**^2^**, Hao Yang**^3^**, Lingyan Zheng**^2^**, Shukai Gu**^2^**, Yang Zhang**^3^**, Wei Hu**^1^**, Fengcheng Li**^4^**^,^*, Haibin Dai**^1,^***, Feng Zhu**^1^**^,^**^2^**^,^*

**^1^** Department of Pharmacy, The Second Affiliated Hospital, Zhejiang University School of Medicine, Hangzhou, 310009, China

**^2^** College of Pharmaceutical Sciences, State Key Laboratory of Advanced Drug Delivery and Release Systems, Zhejiang University, Hangzhou 310058, China

**^3^** School of Pharmacy, Hebei Medical University, Shijiazhuang 050017, China

**^4^** Children’s Hospital, Zhejiang University School of Medicine, National Clinical Research Center for Child Health, Hangzhou 310058, China.

† These authors contributed equally to this work.

* Corresponding Authors: Feng Zhu (zhufeng@zju.edu.cn), Haibin Dai (haibindai@zju.edu.cn) and Fengcheng Li (lifengcheng@zju.edu.cn)

**Supplementary Methods**

**The Formulas of Evaluation Metrics**

Several widely used evaluation metrics were adopted in this study, including accuracy (ACC), precision, recall, area under the receiver operator characteristic curve (AUROC), area under the precision-recall curve (AUPRC), F1 score, Matthews correlation coefficient (MCC), Dice similarity coefficient (DSC) and Brier score. The formulas for computing these metrics are as follows:

$$\begin{aligned} \mathrm{ACC}=\frac{TP+TN}{TP+TN+FP+FN}\#\left( AUTONUM \backslash* Arabic \right) \end{aligned}$$

$$\begin{aligned} \mathrm{Precision}=\frac{\mathrm{TP}}{TP+FP}\#\left( AUTONUM \backslash* Arabic \right) \end{aligned}$$

$$\begin{aligned} \mathrm{Recall}=\frac{\mathrm{TP}}{TP+FN}\#\left( AUTONUM \backslash* Arabic \right) \end{aligned}$$

$$\begin{aligned} F1=\frac{2\times PRE\times REC}{PRE+REC}\#\left( AUTONUM \backslash* Arabic \right) \end{aligned}$$

$$\begin{aligned} \mathrm{MCC}=\frac{TP\times TN-FP\times FN}{\sqrt{\left( TP+FP \right)\times\left( TP+FN \right)\times\left( TN+FP \right)\times\left( TN+FN \right)}}\#\left( AUTONUM \backslash* Arabic \right) \end{aligned}$$

$$\begin{aligned} \mathrm{DSC}=\frac{2\times TP}{\left( TP+FP \right)+\left( TP+FN \right)}\#\left( AUTONUM \backslash* Arabic \right) \end{aligned}$$

$$\begin{aligned} \mathrm{Brier}\mathrm{score}=-\frac{1}{N}\sum_{i=1}^{N} ({\hat{y_{i}}-y_{i})}^{2}\#\left( AUTONUM \backslash* Arabic \right) \end{aligned}$$

where $\mathrm{TP}$, $\mathrm{TN}$, $\mathrm{FP}$ and $\mathrm{FN}$ denote the number of true positives, true negatives, false positives and false negatives, respectively. $y_{i}$  is the true label of residue $i$, $\hat{y_{i}}$ is the predicted probability of residue $i$ being a binding site, and $N$ is the total number of all residues.


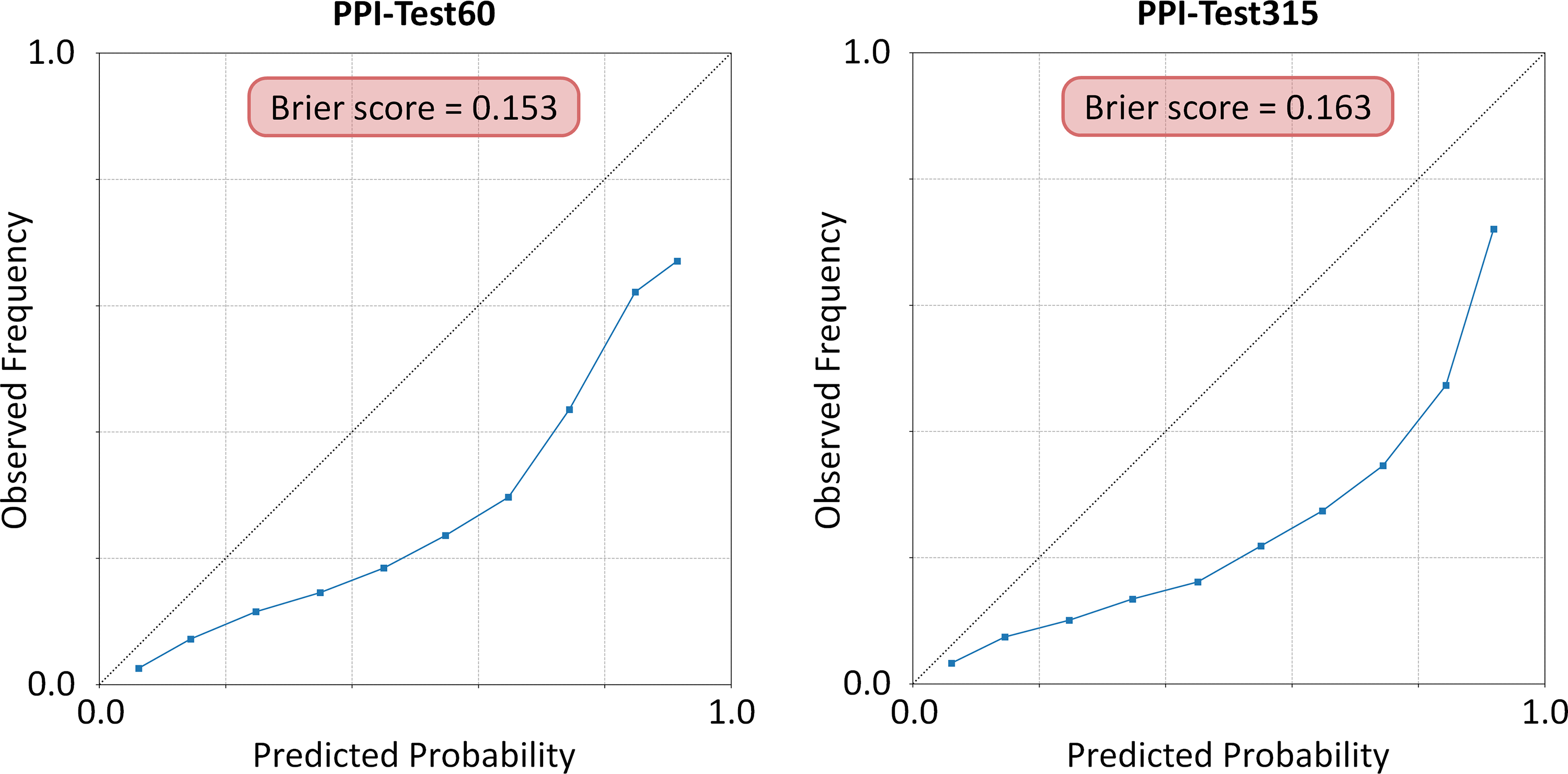


**Figure S1**. Calibration analysis of ALLSites on the PPI-Test60 and PPI-Test315 datasets.


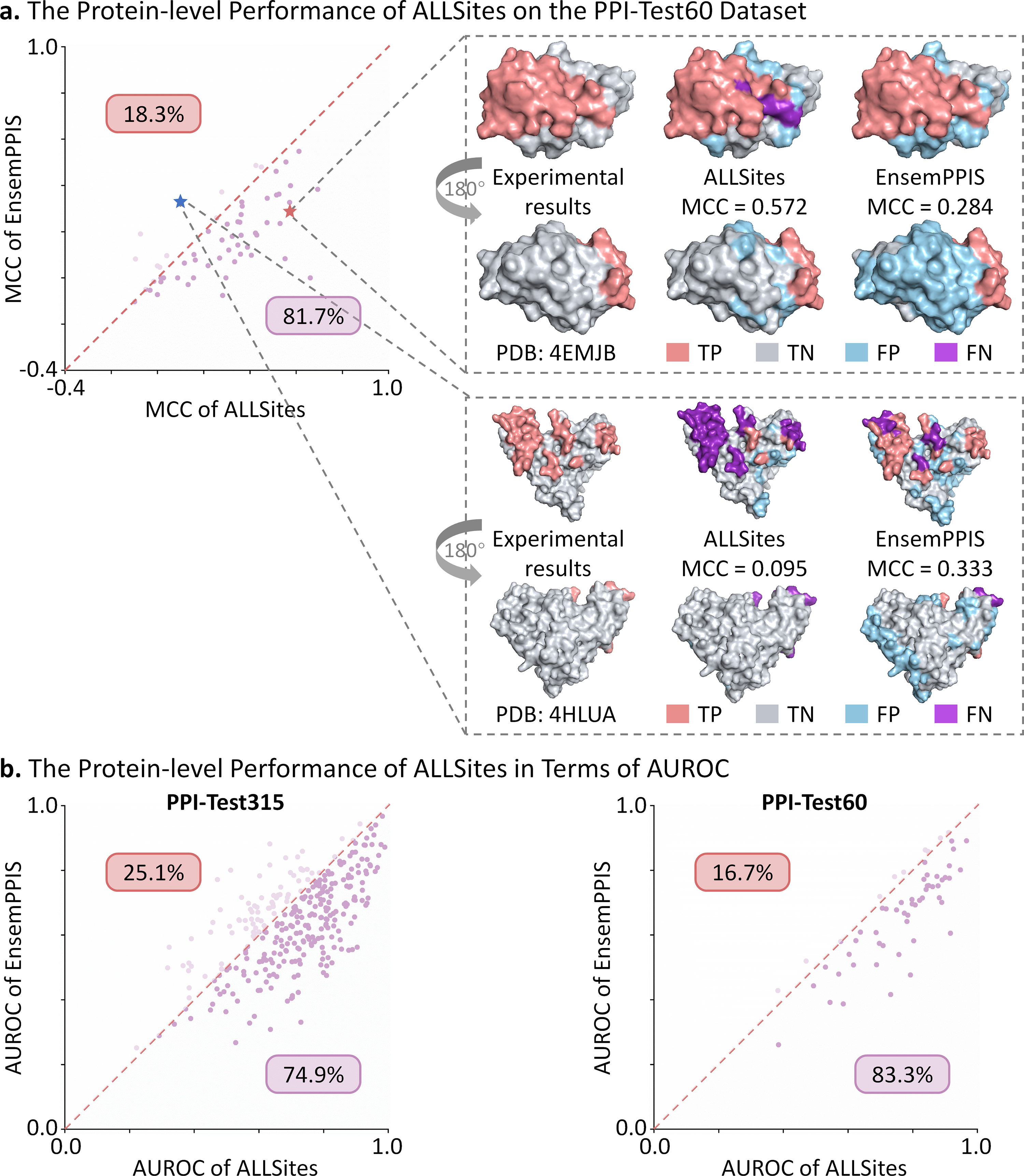


**Figure S2**. The protein-level performance of ALLSites in predicting protein-binding sites. **a.** The protein-level performance of ALLSites on the PPI-Test60 dataset. The MCC metric is calculated for each protein based on ALLSites’ predictions. Two representative proteins (PDB ID: 4EMJB and PDB ID: 4HLUA) are presented to show the predictions of ALLSites and EnsemPPIS alongside the corresponding experimental results. **b.** The protein-level performance of ALLSites on the PPI-Test315 and PPI-Test60 datasets in terms of AUROC.

**Table S1.** Performance evaluation of ALLSites using different protein language models for protein representation on the PPI-Test70, PPI-Test60 and PPI-Terst315 datasets. The best performance for each metric is highlighted in bold.

| **Method** | **ACC** | **AUROC** | **AUPRC** | **F1** | **MCC** |
| --- | --- | --- | --- | --- | --- |
| **PPI-Test70** | | | | | |
| ALLSites-ESM2 | **0.720** | 0.755 | 0.438 | **0.474** | **0.319** |
| ALLSites-ESMC | 0.613 | **0.758** | **0.458** | 0.451 | 0.295 |
| **PPI-Test60** | | | | | |
| ALLSites-ESM2 | **0.818** | **0.781** | **0.436** | **0.448** | **0.340** |
| ALLSites-ESMC | 0.691 | 0.769 | 0.425 | 0.417 | 0.293 |
| **PPI-Test315** | | | | | |
| ALLSites-ESM2 | **0.739** | **0.746** | 0.334 | **0.392** | **0.273** |
| ALLSites-ESMC | 0.629 | 0.738 | **0.335** | 0.360 | 0.241 |

**Table S2.** The source data for benchmark results of PPI-Test60 dataset shown in **Figure 2a**. The best performance for each metric is highlighted in bold and the second-best performance is underlined.

| **Class** | **Method** | **ACC** | **AUROC** | **AUPRC** | **F1** | **MCC** |
| --- | --- | --- | --- | --- | --- | --- |
| Structure-based | SPPIDER ^b^ | 0.735 | 0.511 | 0.193 | 0.220 | 0.062 |
|  | DeepPPISP ^a^ | 0.657 | 0.653 | 0.276 | 0.335 | 0.167 |
|  | GraphPPIS ^a^ | 0.772 | 0.681 | **0.487** | 0.431 | 0.307 |
|  | MaSIF-site ^a^ | 0.780 | 0.775 | 0.439 | 0.446 | 0.326 |
|  | RGN ^a^ | 0.813 | 0.779 | 0.429 | **0.455** | **0.344** |
| Sequence-based | ProNA2020 ^b^ | 0.738 | - | - | 0.326 | 0.176 |
|  | SCRIBER ^b^ | 0.667 | 0.665 | 0.278 | 0.350 | 0.193 |
|  | DLPred ^b^ | 0.700 | 0.677 | 0.294 | 0.357 | 0.205 |
|  | DELPHI ^a^ | 0.706 | 0.699 | 0.319 | 0.373 | 0.227 |
|  | EnsemPPIS ^a^ | 0.716 | 0.726 | 0.352 | 0.401 | 0.266 |
|  | ALLSites | **0.818** | **0.781** | 0.436 | 0.448 | 0.340 |

*Note*: ^a^ Results generated by reproducing the source code. ^b^ Results obtained by using the web server. ProNA2020 only makes binary predictions, and its AUROC and AUPRC are not calculated.

**Table S3.** The source data for benchmark results of PPI-Test315 dataset shown in **Figure 2a**. The best performance for each metric is highlighted in bold and the second-best performance is underlined.

| **Class** | **Method** | **ACC** | **AUROC** | **AUPRC** | **F1** | **MCC** |
| --- | --- | --- | --- | --- | --- | --- |
| Structure-based | GraphPPIS ^a^ | 0.722 | 0.743 | 0.338 | 0.382 | 0.260 |
|  | RGN ^a^ | 0.718 | **0.752** | **0.360** | 0.390 | 0.273 |
| Sequence-based | EnsemPPIS ^a^ | 0.651 | 0.699 | 0.294 | 0.346 | 0.213 |
|  | ALLSites | **0.739** | 0.746 | 0.334 | **0.392** | **0.273** |

*Note*: ^a^ Results generated by reproducing the source code.

**Table S4.** The source data for benchmark results of PepPI-Test125 dataset shown in **Figure 2c**. The best performance for each metric is highlighted in bold and the second-best performance is underlined.

| **Class** | **Method** | **AUROC** | **MCC** |
| --- | --- | --- | --- |
| Structure-based | PepSite | 0.610 | 0.200 |
|  | PeptiMap | 0.630 | 0.270 |
|  | SPRINT-Str | 0.780 | 0.290 |
|  | PepNN-Struct | 0.841 | 0.321 |
| Sequence-based | SPRINT-Seq | 0.680 | 0.200 |
|  | Visual | 0.730 | 0.170 |
|  | PepBind | 0.793 | 0.372 |
|  | PepNN-Seq | 0.805 | 0.278 |
|  | PepBCL | 0.815 | **0.385** |
|  | ALLSites | **0.843** | 0.383 |

*Note*: The performance metrics of competing methods were obtained from the PepBCL literature, given their use of identical data splitting and model training procedures.

**Table S5.** The source data for benchmark results of PepPI-Test639 dataset shown in **Figure 2c**. The best performance for each metric is highlighted in bold and the second-best performance is underlined.

| **Class** | **Method** | **AUROC** | **MCC** |
| --- | --- | --- | --- |
| Structure-based | PepNN-Struct | **0.838** | 0.301 |
| Sequence-based | PepBind | 0.767 | **0.348** |
|  | PepNN-Seq | 0.792 | 0.251 |
|  | PepBCL | 0.804 | 0.312 |
|  | ALLSites | 0.817 | 0.316 |

*Note*: The performance metrics of competing methods were obtained from the PepBCL literature, given their use of identical data splitting and model training procedures.

**Table S6.** The source data for benchmark results of SMPI-Test348 dataset shown in **Figure 3a**. The best performance for each metric is highlighted in bold.

| **Class** | **Method** | **ACC** | **Precision** | **Recall** | **F1** | **MCC** |
| --- | --- | --- | --- | --- | --- | --- |
| Structure-based | P2Rank ^a^ | 0.917 | 0.597 | 0.361 | 0.450 | 0.424 |
| Sequence-based | ALLSites | **0.926** | **0.609** | **0.593** | **0.601** | **0.560** |

*Note*: ^a^ Results generated by reproducing the source code.

**Table S7.** The source data for benchmark results of CarbPI-Test162 dataset shown in **Figure 3a**. The best performance for each metric is highlighted in bold and the second-best performance is underlined.

| **Class** | **Method** | **DSC** | **Precision** | **Recall** | **MCC** |
| --- | --- | --- | --- | --- | --- |
| Structure-based | FTMap | 0.351 | 0.284 | 0.505 | 0.222 |
|  | CAPSIF:G | 0.543 | 0.541 | 0.590 | 0.538 |
|  | CAPSIF:V | 0.597 | 0.598 | 0.647 | 0.599 |
| Sequence-based | ALLSites | **0.609** | **0.624** | **0.652** | **0.603** |

*Note*: The performance metrics of competing methods were obtained from the CAPSIF:V literature, given their use of identical data splitting and model training procedures.

**Table S8.** The source data for benchmark results of DPI-Test129 dataset shown in **Figure 4a**. The best performance for each metric is highlighted in bold and the second-best performance is underlined.

| **Class** | **Method** | **AUROC** | **AUPRC** | **Precision** | **Recall** | **F1** | **MCC** |
| --- | --- | --- | --- | --- | --- | --- | --- |
| Structure-based | COACH-D ^b^ | 0.710 | 0.269 | 0.357 | 0.367 | 0.362 | 0.321 |
|  | NucBind ^b^ | 0.811 | 0.294 | 0.381 | 0.330 | 0.354 | 0.317 |
|  | DNABind ^b^ | 0.858 | 0.402 | 0.346 | 0.601 | 0.440 | 0.411 |
|  | GraphBind ^a^ | 0.927 | **0.519** | **0.425** | **0.676** | **0.522** | **0.499** |
| Sequence-based | SVMnuc ^b^ | 0.812 | 0.302 | 0.371 | 0.316 | 0.341 | 0.304 |
|  | NCBRPred ^b^ | 0.823 | 0.310 | 0.392 | 0.312 | 0.347 | 0.313 |
|  | DNAPred ^b^ | 0.845 | 0.367 | 0.353 | 0.396 | 0.373 | 0.332 |
|  | ALLSites | **0.927** | 0.517 | 0.401 | 0.674 | 0.501 | 0.480 |

*Note*: ^a^ Results generated by reproducing the source code. ^b^ Results obtained by using the web server.

**Table S9.** The source data for benchmark results of ALLSites on DPI-Test129 dataset with ten random runs. The results were presented as mean ± standard deviation.

| **Random** | **AUROC** | **AUPRC** | **Precision** | **Recall** | **F1** | **MCC** |
| --- | --- | --- | --- | --- | --- | --- |
| 1 | 0.931 | 0.523 | 0.342 | 0.775 | 0.475 | 0.471 |
| 2 | 0.928 | 0.522 | 0.380 | 0.709 | 0.495 | 0.479 |
| 3 | 0.926 | 0.505 | 0.389 | 0.660 | 0.490 | 0.467 |
| 4 | 0.926 | 0.525 | 0.448 | 0.613 | 0.518 | 0.489 |
| 5 | 0.924 | 0.513 | 0.395 | 0.684 | 0.501 | 0.481 |
| 6 | 0.928 | 0.511 | 0.402 | 0.671 | 0.503 | 0.481 |
| 7 | 0.927 | 0.518 | 0.463 | 0.591 | 0.519 | 0.489 |
| 8 | 0.927 | 0.521 | 0.395 | 0.679 | 0.499 | 0.478 |
| 9 | 0.928 | 0.511 | 0.406 | 0.674 | 0.507 | 0.485 |
| 10 | 0.927 | 0.518 | 0.394 | 0.688 | 0.501 | 0.481 |
| Average | 0.927  ±0.002 | 0.517  ±0.006 | 0.401  ±0.032 | 0.674  ±0.048 | 0.501  ±0.012 | 0.480  ±0.007 |

**Table S10.** The source data for benchmark results of DPI-Test181 dataset shown in **Figure 4a**. The best performance for each metric is highlighted in bold and the second-best performance is underlined.

| **Class** | **Method** | **AUROC** | **AUPRC** | **Precision** | **Recall** | **F1** | **MCC** |
| --- | --- | --- | --- | --- | --- | --- | --- |
| Structure-based | COACH-D ^b^ | 0.655 | 0.172 | 0.280 | 0.254 | 0.266 | 0.235 |
|  | NucBind ^b^ | 0.796 | 0.191 | 0.248 | 0.293 | 0.269 | 0.234 |
|  | DNABind ^b^ | 0.825 | 0.219 | 0.199 | 0.535 | 0.290 | 0.279 |
|  | GraphBind ^a^ | 0.904 | 0.339 | **0.293** | **0.624** | **0.399** | **0.392** |
| Sequence-based | NCBRPred ^b^ | 0.771 | 0.183 | 0.241 | 0.259 | 0.250 | 0.215 |
|  | DNAPred ^b^ | 0.802 | 0.230 | 0.223 | 0.334 | 0.267 | 0.233 |
|  | SVMMnuc ^b^ | 0.803 | 0.193 | 0.242 | 0.289 | 0.263 | 0.229 |
|  | ALLSites | **0.904** | **0.366** | 0.292 | 0.595 | 0.389 | 0.379 |

*Note*: ^a^ Results generated by reproducing the source code. ^b^ Results obtained by using the web server.

**Table S11.** The source data for benchmark results of ALLSites on DPI-Test181 dataset with ten random runs. The results were presented as mean ± standard deviation.

| **Random** | **AUROC** | **AUPRC** | **Precision** | **Recall** | **F1** | **MCC** |
| --- | --- | --- | --- | --- | --- | --- |
| 1 | 0.907 | 0.365 | 0.230 | 0.725 | 0.349 | 0.365 |
| 2 | 0.906 | 0.366 | 0.280 | 0.632 | 0.388 | 0.384 |
| 3 | 0.902 | 0.364 | 0.290 | 0.577 | 0.386 | 0.373 |
| 4 | 0.906 | 0.368 | 0.324 | 0.538 | 0.404 | 0.384 |
| 5 | 0.901 | 0.366 | 0.292 | 0.589 | 0.390 | 0.379 |
| 6 | 0.904 | 0.361 | 0.299 | 0.575 | 0.393 | 0.379 |
| 7 | 0.904 | 0.369 | 0.342 | 0.48 | 0.406 | 0.381 |
| 8 | 0.901 | 0.358 | 0.292 | 0.597 | 0.392 | 0.381 |
| 9 | 0.903 | 0.367 | 0.285 | 0.600 | 0.386 | 0.376 |
| 10 | 0.907 | 0.372 | 0.288 | 0.620 | 0.393 | 0.386 |
| Average | 0.904  ±0.002 | 0.366  ±0.004 | 0.292  ±0.028 | 0.595  ±0.057 | 0.389  ±0.015 | 0.379  ±0.006 |

**Table S12.** The source data for benchmark results of RPI-Test117 dataset shown in **Figure 4b**. The best performance for each metric is highlighted in bold and the second-best performance is underlined.

| **Class** | **Method** | **AUROC** | **Precision** | **Recall** | **F1** | **MCC** |
| --- | --- | --- | --- | --- | --- | --- |
| Structure-based | COACH-D ^b^ | 0.663 | 0.252 | 0.221 | 0.235 | 0.195 |
|  | NucBind ^b^ | 0.715 | 0.235 | 0.231 | 0.233 | 0.189 |
|  | aaRNA ^b^ | 0.771 | 0.166 | **0.484** | 0.247 | 0.214 |
|  | NucleicNet ^a^ | 0.788 | 0.201 | 0.371 | 0.261 | 0.216 |
|  | GraphBind ^a^ | **0.854** | **0.294** | 0.463 | **0.358** | **0.322** |
| Sequence-based | RNABindRPlus ^b^ | 0.717 | 0.227 | 0.273 | 0.248 | 0.202 |
|  | SVMnuc ^b^ | 0.729 | 0.24 | 0.231 | 0.235 | 0.192 |
|  | ALLSites ^b^ | 0.853 | 0.277 | 0.457 | 0.336 | 0.303 |

*Note*: ^a^ Results generated by reproducing the source code. ^b^ Results obtained by using the web server.

**Table S13.** The source data for benchmark results of ALLSites on RPI-Test117 dataset with ten random runs. The results were presented as mean ± standard deviation.

| **Random** | **AUROC** | **AUPRC** | **Precision** | **Recall** | **F1** | **MCC** |
| --- | --- | --- | --- | --- | --- | --- |
| 1 | 0.851 | 0.273 | 0.229 | 0.569 | 0.326 | 0.304 |
| 2 | 0.857 | 0.263 | 0.316 | 0.361 | 0.337 | 0.297 |
| 3 | 0.854 | 0.254 | 0.244 | 0.533 | 0.335 | 0.307 |
| 4 | 0.852 | 0.242 | 0.286 | 0.419 | 0.340 | 0.300 |
| 5 | 0.855 | 0.255 | 0.319 | 0.350 | 0.334 | 0.294 |
| 6 | 0.854 | 0.271 | 0.342 | 0.332 | 0.337 | 0.299 |
| 7 | 0.851 | 0.249 | 0.233 | 0.561 | 0.329 | 0.305 |
| 8 | 0.851 | 0.244 | 0.274 | 0.461 | 0.344 | 0.307 |
| 9 | 0.857 | 0.255 | 0.282 | 0.461 | 0.350 | 0.313 |
| 10 | 0.848 | 0.251 | 0.243 | 0.524 | 0.331 | 0.303 |
| Average | 0.853  ±0.003 | 0.256  ±0.010 | 0.277  ±0.038 | 0.457  ±0.085 | 0.336  ±0.006 | 0.303  ±0.005 |

**Table S14.** The source data for evaluating the impact of AlphaFold2-predicted structures on GraphBind shown in **Figure 4c**. The best performance for each metric is highlighted in bold and the second-best performance is underlined.

| **Method** | **AUROC** | **AUPRC** | **Precision** | **Recall** | **F1** | **MCC** |
| --- | --- | --- | --- | --- | --- | --- |
| **DPI-Test129** | | | | | | |
| GraphBind ^a^ | 0.927 | **0.519** | **0.425** | **0.676** | **0.522** | **0.499** |
| GraphBind-AF2 ^a^ | 0.916 | 0.497 | 0.434 | 0.625 | 0.512 | 0.484 |
| ALLSites | **0.927** | 0.517 | 0.401 | 0.674 | 0.501 | 0.480 |
| **DPI-Test181** | | | | | | |
| GraphBind ^a^ | 0.904 | 0.339 | **0.293** | **0.624** | **0.399** | **0.392** |
| GraphBind-AF2 ^a^ | 0.893 | 0.317 | 0.304 | 0.505 | 0.380 | 0.357 |
| ALLSites | **0.904** | **0.366** | 0.292 | 0.595 | 0.389 | 0.379 |

*Note:* ^a^ Results generated by reproducing the source code.

**Table S15.** Dataset statistics and loss weights of ALLSites across all binding site prediction tasks.

| **Site type** | **Task** | **Dataset** | **No. proteins** | **No. residues** | **No. binding residues** | **No. non-binding residues** | **Ratio of binding residues (%)** | **Loss weight (non-binding : binding)** |
| --- | --- | --- | --- | --- | --- | --- | --- | --- |
| PPI site | PPI-Train352 and PPI-Test70 | PPI-Train352 | 352 | 73181 | 11079 | 62102 | 15.14 | 1 : 5 |
|  |  | PPI-Test70 | 70 | 11791 | 2332 | 9459 | 19.78 |  |
|  | PPI-Train9982 and PPI-Test355 | PPI-Train9982 | 9982 | 4254198 | 427687 | 3826511 | 10.05 | 1 : 3 |
|  |  | PPI-Test355 | 355 | 95940 | 11467 | 84473 | 11.95 |  |
|  | PPI-Train335, PPI-Test60 and PPI-Test315 | PPI-Train335 | 335 | 66366 | 10374 | 55992 | 15.63 | 1 : 5 |
|  |  | PPI-Test60 | 60 | 13144 | 2075 | 11069 | 15.79 |  |
|  |  | PPI-Test315 | 315 | 65331 | 9355 | 55976 | 14.32 |  |
| PepPI site | PepPI-Train1154 and PepPI-Test125 | PepPI-Train1154 | 1154 | 276822 | 15030 | 261792 | 5.43 | 1 : 1 |
|  |  | PepPI-Test125 | 125 | 30870 | 1719 | 29151 | 5.57 |  |
|  | PepPI-Train640 and PepPI-Test639 | PepPI-Train640 | 640 | 157362 | 8259 | 149103 | 5.25 | 1 : 5 |
|  |  | PepPI-Test639 | 639 | 150330 | 8490 | 141840 | 5.65 |  |
| SMPI site | SMPI-Train1628, SMPI-Valid348 and SMPI-Test348 | SMPI-Train1628 | 1628 | 678373 | 61210 | 617163 | 9.02 | 1 : 1 |
|  |  | SMPI-Valid348 | 348 | 138926 | 13004 | 125922 | 9.36 |  |
|  |  | SMPI-Test348 | 348 | 140821 | 13171 | 127650 | 9.35 |  |
| CarbPI site | Carb-Train517, Carb-Valid129 and Carb-Test162 | Carb-Train517 | 517 | 186665 | 7207 | 179458 | 3.86 | 1 : 3 |
|  |  | Carb-Valid129 | 129 | 49680 | 1815 | 47865 | 3.65 |  |
|  |  | Carb-Test162 | 162 | 56928 | 2237 | 54691 | 3.93 |  |
| DPI site | DPI-Train573, DPI-Test129 and DPI-Test181 | DPI-Train573 | 573 | 159883 | 14479 | 145404 | 9.06 | 1 : 3 |
|  |  | DPI-Test129 | 129 | 37515 | 2240 | 35275 | 5.97 |  |
|  |  | DPI-Test181 | 181 | 75258 | 3208 | 72050 | 4.26 |  |
| RPI site | RPI-Train495 and RPI-Test117 | RPI-Train495 | 495 | 136899 | 14609 | 122290 | 10.67 | 1 : 3 |
|  |  | RPI-Test117 | 117 | 37345 | 2031 | 35314 | 5.44 |  |

*Note:* This table provides comprehensive statistics for each dataset including the number of proteins, total residues, binding/non-binding residues, binding ratio, and the loss weight values used during model training to address class imbalance. The loss weights were determined by hyperparameter optimization based on the model’s predictive performance on the validation dataset.

**Table S16.** The sources of the evaluation performance for all baseline methods across the assessed tasks.

| **Site Type** | **Task** | **Obtained using the web server** | **Obtained by reproducing the source code on the same splits** | **Directly collected from the corresponding literature** |
| --- | --- | --- | --- | --- |
| PPI site | PPI-Train352 and PPI-Test70 | SPPIDER, ProNA2020, SCRIBER, DLPred | DeepPPISP, EGRET, SPRINGS, DELPHI, EnsemPPIS | IntPred, ISIS, RF_PPI, PSIVER  (From DeepPPISP) |
|  | PPI-Train9982 and PPI-Test355 | DLPred, SCRIBER | SPRINGS, DELPHI, EnsemPPIS | N.A. |
|  | PPI-Train335, PPI-Test60 and PPI-Test315 | SPPIDER, ProNA2020, SCRIBER, DLPred | DeepPPISP, GraphPPIS, MaSIF-site, RGN, DELPHI, EnsemPPIS | N.A. |
| PepPI site | PepPI-Train1154 and PepPI-Test125 | N.A. | N.A. | PepSite, PeptiMap, SPRINT-Str, PepNN-Struct, SPRINT-Seq, Visual, PepBind, PepNN-Seq, PepBCL  (From PepBCL) |
|  | PepPI-Train640 and PepPI-Test639 | N.A. | N.A. | PepNN-Struct, PepBind, PepNN-Seq, PepBCL (From PepBCL) |
| SMPI site | SMPI-Train1628, SMPI-Valid348 and SMPI-Test348 | N.A. | P2Rank  (load the pre-trained model parameters) | N.A. |
| CarbPI site | Carb-Train517, Carb-Valid129 and Carb-Test162 | N.A. | N.A. | FTMap, CAPSIF:G, CAPSIF:V  (From CAPSIF:V) |
| DPI site | DPI-Train573, DPI-Test129 and DPI-Test181 | COACH-D, NucBind, DNABind, SVMnuc, NCBRPred, DNAPred | GraphBind | N.A. |
| RPI site | RPI-Train495 and RPI-Test117 | COACH-D, NucBind, aaRNA, RNABindRPlus, SVMnuc | NucleicNet, GraphBind | N.A. |
